# A Rare Primary Cutaneous Myoepithelial Carcinoma in the Axilla Accompanied by Lymph Node Metastasis: A Case Report

**DOI:** 10.1002/cai2.157

**Published:** 2024-11-12

**Authors:** Xudong Zhu, Shenglong Li

**Affiliations:** ^1^ Department of General Surgery Cancer Hospital of Dalian University of Technology, Cancer Hospital of China Medical University, Liaoning Cancer Hospital & Institute Shenyang Liaoning China; ^2^ Liaoning Provincial Key Laboratory of Precision Medicine for Malignant Tumors Shenyang Liaoning China; ^3^ Second Ward of Bone and Soft Tissue Tumor Surgery Cancer Hospital of Dalian University of Technology, Cancer Hospital of China Medical University, Liaoning Cancer Hospital & Institute Shenyang Liaoning China

**Keywords:** axilla, cytokeratin, lymph node metastasisp, primary cutaneous myoepithelial carcinoma, smooth muscle actin

## Abstract

Primary cutaneous myoepithelial carcinoma is an extremely rare tumor, and to the best of our knowledge, it has never been reported to occur in the axilla. Furthermore, the pathological and clinical factors of cutaneous myoepithelial carcinoma are poorly understood and may considerably affect prognosis and treatment. Here, we report a case of a 44‐year‐old male patient who was diagnosed with primary cutaneous myoepithelial carcinoma in the axilla accompanied by extensive lymph node metastasis. After an enlarged resection of the left axillary mass, axillary lymph node dissection, and the administration of postoperative chemotherapy and local radiotherapy, there were no signs of tumor recurrence or metastasis. At the time of manuscript preparation, the patient was recurrence‐free. This case may contribute to the clinical management, diagnosis, and treatment of primary cutaneous myoepithelial carcinoma.

## Graphical abstract

Primary cutaneous myoepithelial carcinoma is an extremely rare tumor and has never been reported to occur in the axilla. Furthermore, cutaneous myoepithelial carcinoma is uncommon, so its pathological and clinical factors are poorly understood and may considerately affect prognosis and treatment. Herein, we reported a case of a 44‐year‐old male patient who was diagnosed with primary cutaneous myoepithelial carcinoma in the axilla and was accompanied by extensive lymph node metastasis. Furthermore, after an enlarged resection of the left axillary mass, axillary lymph node dissection, and the administration of postoperative chemotherapy and local radiotherapy, there were no signs of tumor recurrence or metastasis. This case may be used by clinicians and may contribute to the clinical management, diagnosis, and treatment of primary cutaneous myoepithelial carcinoma, as our patient is still recurrence‐free.

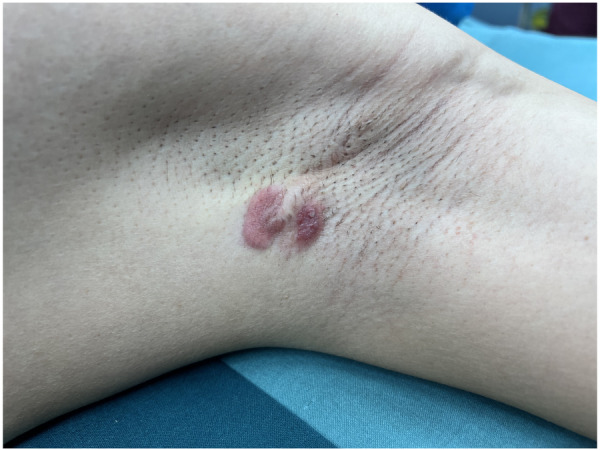

**Abbreviation**
SMA: smooth muscle actin

## 1 Introduction

Malignant myoepithelioma, also known as myoepithelial carcinoma, is a form of rare salivary gland tumor and accounts for approximately 1% of all salivary gland tumors [1]. Primary cutaneous myoepithelial carcinoma is also extremely rare. To our knowledge, no more than 15 cases of primary cutaneous myoepithelial carcinoma have been reported until now [2]. Furthermore, most reported cases not only invaded to the dermis, but also extended subcutaneously, often occurring in the lower extremities, head, and neck areas [3]. The incidence is slightly higher in females than in males. Anatomic, cytological, and stromal characteristics vary among primary cutaneous myoepithelial carcinomas. They may express many myoepithelial markers, such as keratin, smooth muscle actin (SMA), S‐100, calponin, p63, epithelial membrane antigen, and others [4]. There is little information about the pathological and clinical characteristics of primary cutaneous myoepithelial carcinoma, which may affect the prognosis and treatment options. Here, we report a case of a patient who was diagnosed with primary cutaneous myoepithelial carcinoma in the axilla, accompanied by extensive lymph node metastasis.

## 2 Case Presentation

**Figure 1:**
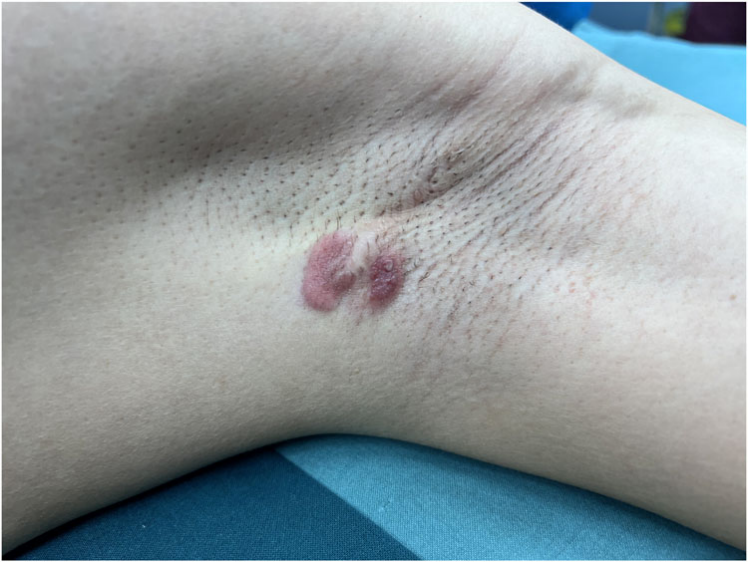
Two masses were observed in the left armpit with local cutaneous redness.

**Figure 2:**
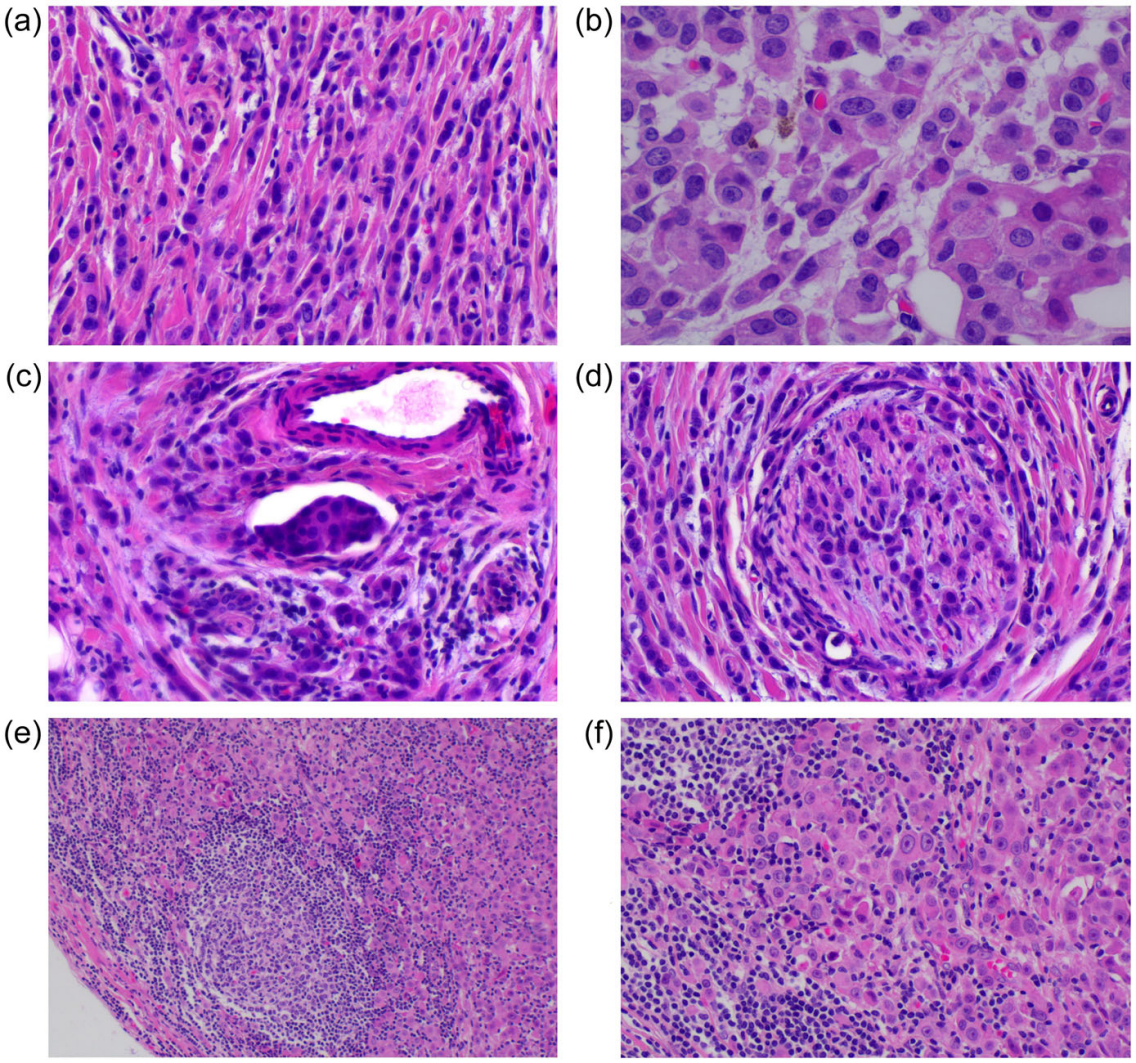
Postoperative pathology confirmed cutaneous myoepithelial carcinoma with lymph node metastasis. (a) Tumor had trabecular infiltrating growth (200×). (b) Tumor cytoplasm was eosinophilic, nucleoli were prominent, and pathological mitotic figures were easily seen (400×). (c) Intravascular tumor thrombus was observed (400×). (d) Neurological invasion was present (400×). (e) Lymph node was invaded by tumor cells (100×). (f) The morphology of the tumor cells in lymph node was consistent with that of the primary lesion, with eosinophilic cytoplasm and significant nucleoli (200×).

**Figure 3:**
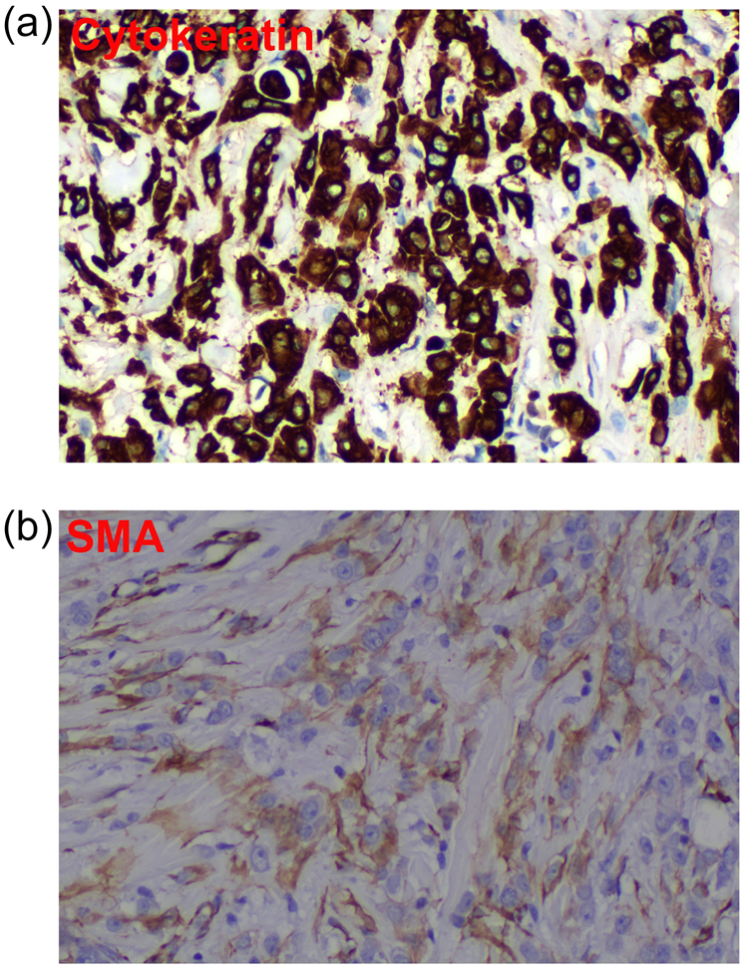
Detection of markers of cutaneous myoepithelial carcinoma by immunochemistry analysis. (a) Positive expression of cytokeratin (200×). (b) Positive expression of SMA (200×). SMA, smooth muscle actin.

A 44‐year‐old male patient had a left axillary mass for 1 year and palpable enlarged lymph nodes in the axilla. On physical examination, two masses were observed in the left axilla, the larger of which was approximately 3 cm × 1 cm, with local cutaneous redness (Figure 1). Multiple nodular shadows with low signal on T1WI and high signal on T2WI were visible on magnetic resonance imaging, with obvious enhancement on T2WI. Other physical examination results and tumor marker levels were normal. The patient underwent an enlarged resection of the left axillary mass and axillary lymph node dissection. Postoperative pathology confirmed a rare cutaneous myoepithelial carcinoma accompanied by lymph node metastasis. A pathological examination revealed trabecular infiltrating growth (Figure 2a), eosinophilic cytoplasm, prominent nucleoli, and mitotic figures visible in the tumor (Figure 2b). Intravascular tumor thrombus and neurological invasion were also found (Figure 2c,d). Metastatic carcinoma 15/17 was also observed in the left axillary lymph node, with the normal structures of the lymph node replaced by tumor cells showing extensive infiltration and growth (Figure 2e). The tumor cells displayed significant nucleoli and eosinophilic cytoplasm similar to the primary lesion (Figure 2f). Furthermore, the tumor cells were positive for expression of cytokeratin and SMA (Figure 3). Postoperative chemotherapy and local radiotherapy were administered, and there have been no signs of tumor recurrence or metastasis as of the time of manuscript preparation. The detailed regimen of postoperative chemotherapy and local radiotherapy was: paclitaxel liposome 270‐mg D1, cisplatin 60‐mg D1, D2, six cycles with local emphasis radiotherapy, 60 grey. The genetic sequencing data of this patient is also supplied as supplementary material (Supporting information S1: Table S1).

## 3 Discussion

The salivary glands, breasts, skin, and respiratory tract are mainly composed of healthy myoepithelial cells that originate from the ectoderm [5]. These myoepithelial cells can transform into rare tumor cells via malignant proliferation [6]. There are very few cases of primary cutaneous myoepithelial carcinoma worldwide. The reported pathogenic sites of origin were mainly the cheek, retro‐auricular region, back, scalp, femoral region, and groin [2, 3, 7, 8, 9, 10]. Primary cutaneous myoepithelial carcinoma that occurs in the axilla and is accompanied by extensive lymph node metastasis has not been previously reported to the best of our knowledge, and thus our team is the first to report this kind of case. Clinicians have little knowledge of diagnosis, pathological conditions, and effective treatments for this type of disease due to its rarity. At present, the most routine treatment strategies reported in the literature were the excision of the tumor and dissection of the lymph nodes if they were involved [1]. Other cases also received carboplatin and paclitaxel chemotherapy, palliative radiation or were enrolled in the phase 1 clinical trial of a checkpoint kinase 1 inhibitor [3, 7]. As a result of its extreme rarity, there is no standard treatment method for primary cutaneous myoepithelial carcinoma. In this case report, we hope to make a clinical contribution to the management of primary cutaneous myoepithelioma.

In this case, we observed two masses in the left axilla with local cutaneous redness. A postoperative pathology report confirmed a rare cutaneous myoepithelial tumor that had metastasized to numerous lymph nodes. Just like the reported pathological morphology, these tumor cells demonstrated infiltrative borders, pleomorphic nuclei, and prominent nucleoli. Additionally, the cytoplasm was eosinophilic and abundant [3]. A similar morphology in the lymph nodes was observed as in the primary tumor. Currently, this patient has not experienced tumor recurrence or metastasis. To our knowledge, almost all patients with primary cutaneous myoepithelial carcinoma could develop local tumor recurrence or distant organ metastasis and have extremely poor survival outcomes, even if they have undergone complete resection of the tumor. Regular postoperative follow‐up and close monitoring must continue to be performed, even though the mass in this case was completely removed and no recurrence or metastasis has been observed.

## Author Contributions

**Xudong Zhu:** conceptualization (equal), data curation (equal), funding acquisition (equal), investigation (equal), software (equal), writing – original draft (equal), writing – review and editing (equal). **Shenglong Li:** conceptualization (equal), data curation (equal), formal analysis (equal), investigation (equal), methodology (equal); software (equal), supervision (equal), writing – original draft (equal), writing – review and editing (equal).

## Ethics Statement

The authors have nothing to report.

## Consent

Written informed consent was obtained from the patient and is uploaded as an additional file for review.

## Conflicts of Interest

The authors declare no conflicts of interest.

## Supporting information

Supporting information.

## Data Availability

The data and materials can be available from the corresponding author for rational reasons.
